# Long-acting capsid inhibitor protects macaques from repeat SHIV challenges

**DOI:** 10.1038/s41586-021-04279-4

**Published:** 2021-12-07

**Authors:** Samuel J. Vidal, Elena Bekerman, Derek Hansen, Bing Lu, Kelly Wang, Judy Mwangi, William Rowe, Federico Campigotto, Jim Zheng, Darryl Kato, Abishek Chandrashekar, Julia Barrett, Shivani Patel, Huahua Wan, Tochi Anioke, Noe B. Mercado, Joseph P. Nkolola, Melissa J. Ferguson, William J. Rinaldi, Christian Callebaut, Wade Blair, Tomas Cihlar, Romas Geleziunas, Stephen R. Yant, Dan H. Barouch

**Affiliations:** 1 Center for Virology and Vaccine Research, Beth Israel Deaconess Medical Center, Harvard Medical School, Boston, MA USA; 2 Division of Infectious Diseases, Brigham and Women’s Hospital and Massachusetts General Hospital, Harvard Medical School, Boston, MA USA; 3 Gilead Sciences, Foster City, CA USA; 4 Alpha Genesis, Inc., Yemassee, SC USA; 5 Ragon Institute of MGH, MIT and Harvard, Cambridge, MA USA

**Keywords:** Retrovirus, Drug development

## Abstract

Because no currently available vaccine can prevent HIV infection, pre-exposure prophylaxis (PrEP) with antiretrovirals (ARVs) is an important tool for combating the HIV pandemic^1,2^. Long-acting ARVs promise to build on the success of current PrEP strategies, which must be taken daily, by reducing the frequency of administration^3^. GS-CA1 is a small-molecule HIV capsid inhibitor with picomolar antiviral potency against a broad array of HIV strains, including variants resistant to existing ARVs, and has shown long-acting therapeutic potential in a mouse model of HIV infection^4^. Here we show that a single subcutaneous administration of GS-CA1 provides long-term protection against repeated rectal simian–human immunodeficiency virus (SHIV) challenges in rhesus macaques. Whereas all control animals became infected after 15 weekly challenges, a single 300 mg kg^−^^1^ dose of GS-CA1 provided per-exposure infection risk reduction of 97% for 24 weeks. Pharmacokinetic analysis showed a correlation between GS-CA1 plasma concentration and protection from SHIV challenges. GS-CA1 levels greater than twice the rhesus plasma protein-adjusted 95% effective concentration conferred 100% protection in this model. These proof-of-concept data support the development of capsid inhibitors as a novel long-acting PrEP strategy in humans.

## Abstract (web summary)

A single dose of a small-molecule HIV capsid inhibitor provides long-term protection from repeated simian–human immunodeficiency virus challenges in macaques and might serve as a novel strategy for HIV prevention in humans.

## Main

The HIV pandemic is a leading cause of morbidity and mortality worldwide^5^. Current strategies for HIV prevention include public health measures as well as vaccine development and improved pre-exposure prophylaxis (PrEP) uptake. Studies conducted by the Centre for the AIDS Programme of Research in South Africa (CAPRISA; trial 004)^6^, Pre-exposure Prophylaxis Initiative (iPrEx)^7^ and Partners PrEP^8^ have shown that tenofovir-based PrEP can reduce HIV transmission. Recent real-world data confirm a significant population-level reduction in HIV-1 incidence in areas in which PrEP uptake is high^9,10^. However, PrEP strategies reliant on frequent drug administration are limited by adherence, which reduces their real-world impact on HIV transmission^11–13^. Long-acting PrEP agents may reduce the barriers associated with daily drug administration, frequent healthcare interactions and the stigma surrounding sexually transmitted infections including HIV^3^. As part of this approach, a long-acting formulation of the integrase strand-transfer inhibitor cabotegravir (CAB-LA), which is injected subcutaneously every 2 months, was shown to reduce HIV transmission in an HIV Prevention Trials Network (HPTN) study (HPTN 083)^14^.

The HIV capsid protein has multiple essential roles in the early and late stages of the viral replication cycle, making it an attractive target for antiretrovirals (ARVs)^15^. Lenacapavir (LEN, formerly GS-6207) is the first clinically validated HIV capsid inhibitor and displays picomolar antiviral activity against both wild-type virus and variants resistant to current ARVs^16^. LEN binds at a highly conserved interface between capsid protein monomers, which causes defects in capsid nuclear import, reduced virion production and aberrant capsid assembly. A long-acting formulation of LEN has been shown to have potent antiviral activity with a maximum 2.3 log_10_ decline in HIV-1 RNA after 9 days of monotherapy^17^ and the potential for twice-yearly subcutaneous dosing in a phase 1b study^18^. GS-CA1, a structural analogue of LEN, has the same capsid-dependent multistage mechanism of action, similar binding affinity for different forms of HIV capsid (i.e., precursor, monomer, pentamer and hexamer), similar potency against both HIV and simian immunodeficiency virus (SIV), and a similar resistance profile. In addition, it has previously been demonstrated to have high preclinical efficacy in a humanized mouse model of HIV-1 treatment (Extended Data Table 1)^4^. However, long-term prophylactic efficacy of LEN or GS-CA1 has not previously been demonstrated. In this study, we assess the potential of a single dose of long-acting capsid inhibitor to offer protection against repeated challenges with simian–human immunodeficiency virus (SHIV) in rhesus macaques. GS-CA1 was chosen for this analysis because of its predicted higher rate of metabolic clearance in comparison to LEN and the associated accelerated washout phase after dose administration, which enables timely evaluation of the prophylactic efficacy of this compound class over a wide range of exposures.

### GS-CA1 inhibits SHIV in macaque cells

**Fig. 1:**
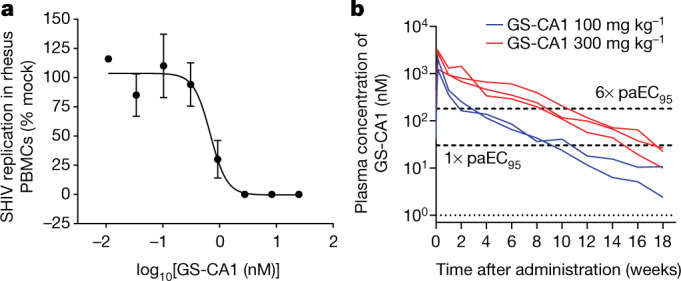
GS-CA1 exhibits potent antiviral activity in vitro and long-acting pharmacokinetics in rhesus macaques. **a**, Representative antiviral dose–response curve for GS-CA1 in rhesus PBMCs acutely infected with SHIV-SF162P3. Data are shown as mean ± s.d. from one of seven assays (*n* = 3 biological replicates each). **b**, Plasma GS-CA1 levels measured by mass spectrometry following a single subcutaneous administration of GS-CA1 dosed at 300 mg kg^−^^1^ in three male rhesus macaques and at 100 mg kg^−^^1^ in two male rhesus macaques. The bottom dotted line represents the assay limit of detection (LOD; 1 nM). The top dashed lines represent one and six times the rhesus paEC_95_ for GS-CA1 (30.2 nM and 181.2 nM, respectively). Source data

GS-CA1 displayed potent in vitro anti-SHIV activity in peripheral blood mononuclear cells (PBMCs) isolated from three individual rhesus macaques of Indian origin (*Macaca mulatta*), with a mean 50% effective concentration (EC_50_) of 0.72 nM (Fig. 1a and Extended Data Table 2). This compound also showed a mean Hill slope value of 3.0 ± 0.7 in high-density antiviral dose–response curves measured against the HIV-1 IIIb strain in MT-4 cells, yielding a calculated 95% effective concentration (EC_95_) of 1.91 nM when applied to the EC_50_ measured in SHIV-infected rhesus PBMCs. In vivo application showed that a large portion of the subcutaneously administered GS-CA1 became bound by plasma proteins, leaving less free drug available for antiviral effects. Competitive equilibrium dialysis was thus used to account for rhesus plasma protein binding to GS-CA1, resulting in a projected 15.8-fold decrease in free GS-CA1 concentration in vivo and yielding a rhesus protein-adjusted EC_95_ (paEC_95_) value of 30.2 nM.

### GS-CA1 shows long-acting plasma exposure

Low hepatic clearance is an essential attribute for a long-acting agent. Titration with ^3^H-labelled GS-CA1 was necessary to accurately measure the low turnover of GS-CA1 in primary rhesus hepatocytes and showed a predicted rate of hepatic clearance of 0.07 l h^−1^ kg^−1^, or 2.9% of the hepatic extraction. These in vitro data suggest that GS-CA1 has the potential to sustain long-acting plasma exposure in rhesus macaques. To test this hypothesis and to select an appropriate GS-CA1 dose for the rhesus efficacy studies, we performed a pilot pharmacokinetic study with a single subcutaneous administration of a GS-CA1 formulation at two dose levels predicted to cover a broad range of plasma exposures over the projected length of the study. GS-CA1 was administered at 100 mg kg^−1^ and 300 mg kg^−1^ to two and three naive rhesus macaques of Indian origin, respectively, and its levels were monitored for 18 weeks. Plasma drug levels peaked in the concentration range of 1–3 µM by day 1 after dose administration, before decreasing to 0.4–1.1 µM by day 7 after dose administration. After this, GS-CA1 levels were maintained in excess of the rhesus paEC_95_ value for at least 8 weeks and 14 weeks and in excess of six times the rhesus paEC_95_ value for at least 1 week and 8 weeks for the 100 mg kg^−1^ and 300 mg kg^−1^ doses, respectively (Fig. 1b). Given that the mean target clinical exposure of LEN for HIV treatment is six times its paEC_95_ in humans^18^, GS-CA1 doses of 300 mg kg^−1^ and 150 mg kg^−1^ were selected for the repeat SHIV challenge study to assess the reduction in transmission risk across rhesus-equivalent GS-CA1 exposures in excess of, equal to and below this clinically relevant target concentration.

### GS-CA1 provides protection from SHIV challenges

**Fig. 2:**
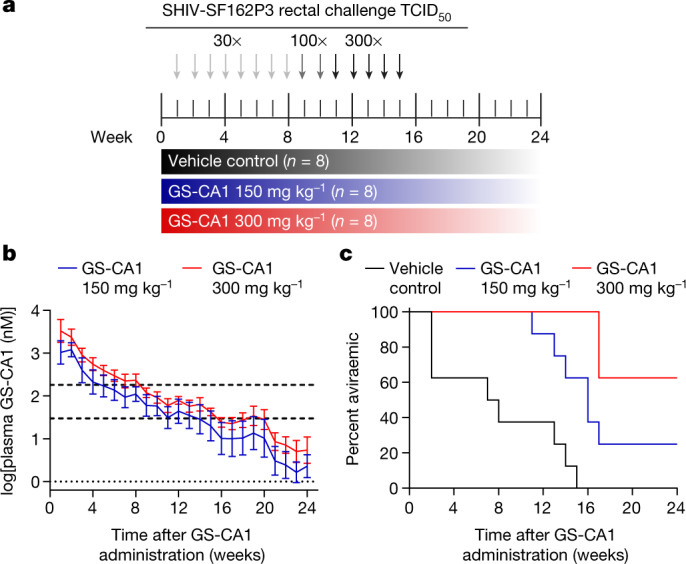
Single-dose GS-CA1 confers long-term protection from repeated rectal SHIV challenge in rhesus macaques. **a**, Study design. Rhesus macaques of Indian origin were treated with a single subcutaneous administration of vehicle control or GS-CA1 at week 0 followed by weekly intrarectal challenges with SHIV-SF162P3 in a dose-escalation scheme until all control animals were confirmed to be infected on week 15. **b**, Plasma GS-CA1 levels measured by mass spectrometry over time in rhesus macaques dosed once at 150 mg kg^−1^ or 300 mg kg^−1^. The data for each group are shown as mean ± s.d. from eight rhesus macaques. The bottom dotted line represents the assay LOD (1 nM). The top dashed lines represent one and six times the rhesus paEC_95_ for GS-CA1 (30.2 nM and 181.2 nM, respectively). **c**, Kaplan–Meier plots showing the development of viraemia as assessed by RT–qPCR for plasma SHIV *gag* among rhesus macaques treated with a single subcutaneous administration of vehicle control (*n* = 8), GS-CA1 dosed at 150 mg kg^−1^ (*n* = 8) or GS-CA1 dosed at 300 mg kg^−1^ (*n* = 8). Source data

We next conducted a study to evaluate the protective efficacy of a single administration of GS-CA1 against repeated, escalating-dose rectal SHIV-SF162P3 challenges in rhesus macaques (Fig. 2a). For this challenge study, 24 rhesus macaques of Indian origin were divided into 3 groups of 8 monkeys each with balanced sex and weight distributions. All animals received a single subcutaneous administration at week 0 in the scapular region. Animals in group 1 received the vehicle control, whereas those in groups 2 and 3 received GS-CA1 doses of 150 mg kg^−1^ and 300 mg kg^−1^, respectively. Consistent with the pilot study, a single administration of GS-CA1 at both 150 mg kg^−1^ and 300 mg kg^−1^ achieved long-acting exposure. Specifically, peaks were reached at plasma GS-CA1 concentrations of 3.0 µM and 5.5 µM approximately 24 h and 42 h after dose administration, respectively, and GS-CA1 levels decreased slowly thereafter with a mean half-life of 287–317 h (Fig. 2b and Extended Data Table 3). The group receiving a dose of 300 mg kg^−1^ remained above the rhesus paEC_95_ and six times above the rhesus paEC_95_ for 14–16 weeks and 5–8 weeks, respectively, whereas the group receiving a dose of 150 mg kg^−1^ remained above these target concentrations for 8–15 weeks and 3–7 weeks, respectively. The variance in GS-CA1 pharmacokinetic parameters across animals was comparable to that observed with 900 mg of LEN in humans^18^, whereas the half-life and length of exposure above the corresponding 6× paEC_95_ threshold were lower than expected.

**Extended Data Fig. 1:**
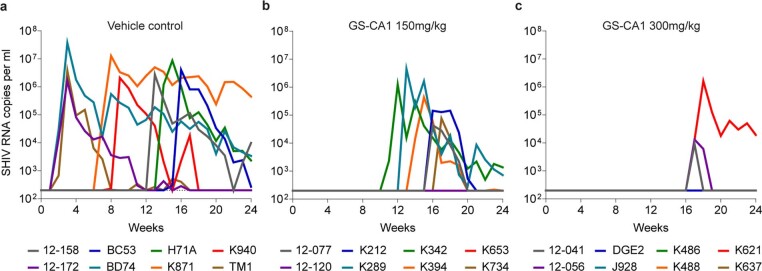
Viral loads among vehicle- and GS-CA-1-treated animals during the SHIV challenge study. **a**, Plasma SHIV viral loads measured by *gag* RT-qPCR among vehicle control rhesus macaques (n = 8) through week 24 following 15 weekly intrarectal challenges. **b**, Same data as (**a**) among rhesus macaques (n = 8) treated with a single 150 mg/kg subcutaneous injection of GS-CA1 at week 0. **c**, Same data as (**a**) and (**b**) among rhesus macaques (n = 8) treated with a single 300 mg/kg subcutaneous injection of GS-CA1 at week 0. Dotted lines represent the assay LOD (200 copies per ml).

To define the protective efficacy of GS-CA1, all animals received 15 weekly intrarectal SHIV-SF162P3 challenges in a dose-escalation protocol beginning at week 1 following GS-CA1 administration. Infection was assessed by quantifying viral *gag* RNA levels in the plasma by quantitative PCR with reverse transcription (RT–qPCR). To define the drug levels required for protection, we monitored plasma viraemia up to week 24 as GS-CA1 levels declined below therapeutic concentrations. Eight weekly intrarectal challenges at 30 times the median tissue culture infectious dose (TCID_50_) resulted in infection of five of the eight vehicle-treated animals (Fig. 2c, Table 1 and Extended Data Fig. 1). Viral challenge dose escalation to 100 TCID_50_ for 2 weeks yielded no additional infections, whereas further escalation to 300 TCID_50_ resulted in infection of the remaining three control animals by week 15.

**Table 1:** Statistical analysis of the rhesus macaque challenge study

	Proportion of animals protected	Median weeks to viraemia (95% CI)	Hazard ratio (95% CI)/per-exposure risk reduction	*P* value
Vehicle control	0/8	7.5 (2, 14)	1	–
GS-CA1, 150 mg kg^−^^1^	2/8	16 (11, –)	0.127 (0.03–0.51)/87%	0.0038
GS-CA1, 300 mg kg^−^^1^	5/8	Not reached (17, –)	0.033 (0.0059–0.188)/97%	0.0001

Proportion of animals protected, median time (in weeks) to viraemia, estimated hazard ratios from the Cox proportional hazard model and corresponding *P* values comparing animals receiving vehicle control and GS-CA1 are shown. CI, confidence interval.

**Extended Data Fig. 2:**
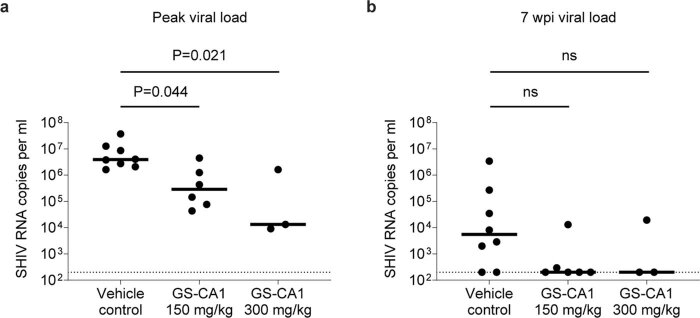
Peak and setpoint SHIV viral loads among infected rhesus macaques treated with vehicle control or GS-CA1. **a**, Peak plasma SHIV viral loads measured by *gag* RT-qPCR among infected rhesus macaques treated with vehicle control (n = 8), GS-CA1 dosed at 150 mg/kg (n = 6), or GS-CA1 dosed at 300 mg/kg (n = 3). **b**, Plasma SHIV viral loads 7 weeks post-infection (wpi) among the same groups as (**a**). Dotted lines represent the assay LOD (200 copies per ml). Solid lines represent group medians. P values reflect Kruskal-Wallis tests.

In contrast to the vehicle-treated group, the group receiving 300 mg kg^−1^ GS-CA1 showed no viraemia until week 17 with this escalating-dose challenge protocol, when three of the eight monkeys became SHIV positive (Fig. 2c, Table 1 and Extended Data Fig. 1). The other five monkeys remained aviraemic until the end of the study, which translates to a 97% per-exposure risk reduction with the 300 mg kg^−1^ dose (*P* = 0.0001, Cox proportional hazard regression analysis). The group that received a single administration of GS-CA1 at the reduced dose of 150 mg kg^−1^ also exhibited fewer and delayed infections relative to the vehicle-treated control group. Specifically, no viraemia was detected until week 11, and two of the eight monkeys remained aviraemic until the end of the study, which represents an 87% per-exposure risk reduction (*P* = 0.0038). The median time to viraemia was 7.5 weeks in the vehicle-treated group and 16 weeks in the group receiving 150 mg kg^−1^ GS-CA1; this point was not reached in the group receiving 300 mg kg^−1^ GS-CA1 owing to an insufficient number of infections. The peak viral loads were significantly lower and the viral loads measured 7 weeks after infection showed a trend towards lower levels among the GS-CA1-treated animals as compared with the vehicle-treated control animals. This result could reflect a residual antiviral effect from subtherapeutic inhibitor levels (Extended Data Fig. 2).

**Extended Data Fig. 3:**
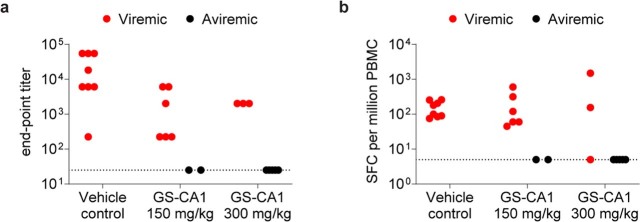
GS-CA1-treated rhesus macaques that remain aviremic do not show immunologic evidence of SHIV infection. **a**, Week 24 anti-gp140 Env ELISA comparing viremic and aviremic animals in the vehicle control and GS-CA1 groups. Dotted line represents assay LOD (1:25 endpoint titer). **b**, Week 19 Gag ELISPOT comparing viremic and aviremic animals in the vehicle control and GS-CA1 groups. Data are represented as spot-forming cells (SFC) per 10^6^ PBMCs. Dotted line represents the assay LOD (5 SFC per million PBMCs).

We next performed immunological analyses in the GS-CA1-treated rhesus monkeys. First, we assessed the development of humoral immune responses against the SHIV envelope (Env) glycoprotein by enzyme-linked immunosorbent assay (ELISA). We detected binding antibody responses to Env at week 24 in all animals with SHIV viraemia but none in those without SHIV viraemia (Extended Data Fig. 3a). Second, we assessed the development of cellular immune responses against SHIV Gag polyprotein by enzyme-linked immune absorbent spot (ELISPOT) assay. We detected T cell responses to the Gag protein at week 19 in all but one animal with SHIV viraemia and in none of the animals without SHIV viraemia (Extended Data Fig. 3b). These immunologic data suggest that the GS-CA1-treated animals that remained aviraemic during the study period were in fact protected from SHIV challenge.

Finally, we performed additional studies to confirm that our plasma viraemia measurements provided early detection of initial infection in the setting of GS-CA1 prophylaxis.

First, we performed intact proviral DNA analysis (IPDA) to detect integrated SHIV in a subset of infected animals treated with vehicle control or GS-CA1 with available PBMCs. Intact SHIV proviruses became detectable at the same time points at which plasma viraemia was detected in all cases except for a single GS-CA1-treated animal (K394) that showed very low-level intact provirus 1 week before detection of viraemia (Extended Data Table 4). Second, we determined the Env ELISA seroconversion time points for all infected animals and observed that viraemia preceded seroconversion in all cases (Extended Data Table 5). The median time to seroconversion from the onset of viraemia was 2.5 weeks (range, 1 to 13 weeks) in vehicle-treated control animals and 4 weeks (range, 1 to 5 weeks) in GS-CA1-treated animals, although the difference between the groups was not significant (*P* = 0.80, Mann–Whitney *U* test).

### Protective levels of GS-CA1 in macaques

**Fig. 3:**
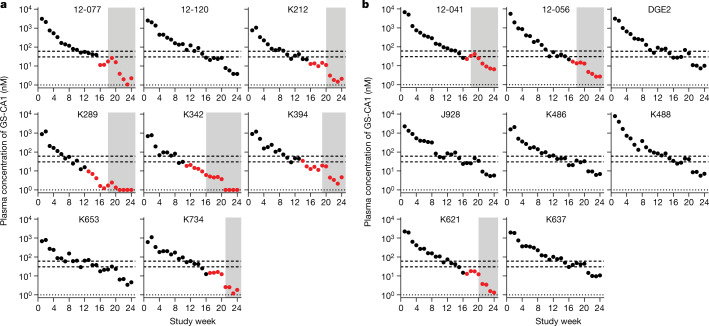
Association between plasma GS-CA1 and protection from SHIV challenge. **a**, Plasma levels of GS-CA1 measured by mass spectrometry among individual rhesus macaques (*n* = 8) treated with a 150 mg kg^−1^ dose over time. Black circles represent time points before the first detection of viraemia. Red circles represent time points including and after the first detection of viraemia. Grey shaded boxes represent serum Env ELISA seroconversion time points. The bottom dotted line represents the assay LOD (1 nM). The bottom and top dashed lines represent one and two times the rhesus paEC_95_, respectively. **b**, Same data as in **a** for rhesus macaques treated with GS-CA1 at a dose of 300 mg kg^−1^. No signal above the assay LOD was observed among the eight placebo-treated control animals throughout the study. Source data

We next investigated the relationship between GS-CA1 plasma concentrations and protection against SHIV challenge. To facilitate exposure comparisons with other antivirals including LEN, we converted the concentrations for GS-CA1 to multiples of its rhesus paEC_95_. To estimate the protective levels for GS-CA1, we focused on six animals that developed viraemia in the group receiving a dose of 150 mg kg^−1^ and three animals that developed viraemia in the group receiving a dose of 300 mg kg^−1^. Assuming a 2-week delay between rectal SHIV infection and detectable peripheral blood viraemia, we averaged the GS-CA1 exposure values 2 weeks before the first detectable viraemia among the infected animals. We estimated that mucosal infection occurred in the presence of 31.4 nM GS-CA1 on average, which is equal to the concentration of rhesus paEC_95_ at a multiple of 1.0 (range, 0.4–1.6; Fig. 3a, b). Therefore, we estimated that, overall, all animals were fully protected in this model when the GS-CA1 plasma concentrations were more than twice the rhesus paEC_95_ value for GS-CA1.

### Plasma virus resistance analysis

**Extended data Fig. 4:**
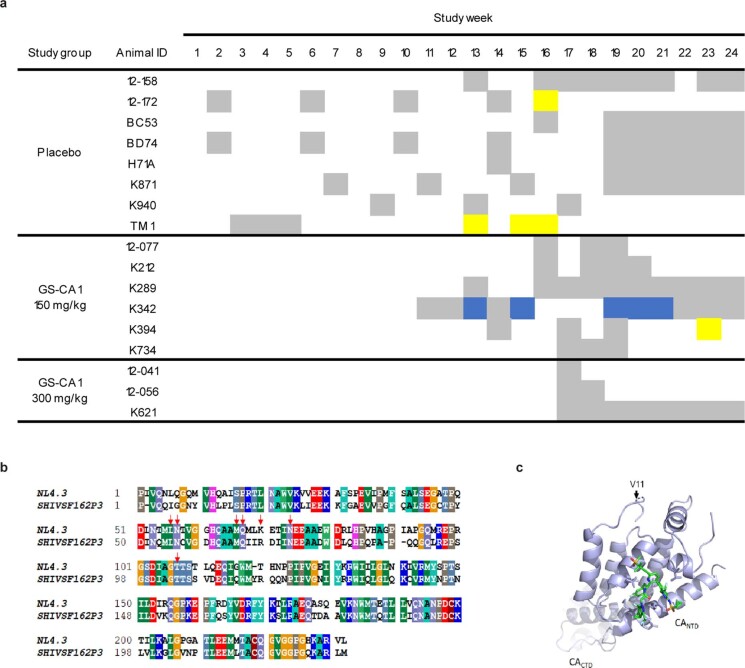
Sequence analysis of study-emergent CA mutations. **a**, Longitudinal CA sequence analysis of select plasma samples from viremic study animals. Samples with population-scale wild-type and V11A CA sequences are shaded grey and blue, respectively, whereas yellow boxes denote sequence failures. **b**, Alignment of HIV-1 (NL4.3 strain) and SHIV-SF162P3 CA amino-acid sequences. Shaded regions represent invariant residues, hashed lines denote sequence gaps, and red arrows highlight HIV-1 residues associated with GS-CA1 resistance (L56, N57, M66, Q67, K70, N74, T107). **c**, A single CA monomer (rendered as ribbons in cyan) within the HIV-1 CA hexamer-LEN crystal structure^16^, with notation of the CA N-terminal and C-terminal domains (CA_NTD_ and CA_CTD_, respectively) and a GS-CA1 molecule (carbon atoms shown as green) docked at the LEN binding site. Location of the only CA variant (V11A) transiently detected by bulk sequencing is shown relative to all HIV-1 CA residues associated with bona fide in vitro GS-CA1 and LEN resistance (highlighted as sticks).

To evaluate potential development of resistance against GS-CA1, particularly during the washout phase, we conducted longitudinal population-level sequence analysis of the SHIV *gag* region encoding capsid in rhesus plasma (Extended Data Fig. 4). As expected, plasma virus obtained from placebo-treated control animals encoded only wild-type capsid protein. Of the plasma virus samples analysed from the nine viraemic animals dosed with GS-CA1, 34 of 35 (97%) produced high-quality sequence reads, with wild-type capsid detected in all nine animals at the end of the study. Animal K342 in the low-dose (150 mg kg^−1^ GS-CA1) group showed transient prevalence of a capsid variant with substitution to alanine at Val11 (V11A; the numbering used here follows the HIV-1 HXB2 reference sequence) between study weeks 13 and 21. This V11A substitution, which is located far outside the GS-CA1-binding site in the capsid, disappeared by week 22 in this animal and is not associated with GS-CA1 resistance^4^. Thus, no study animal showed emergence of variants associated with GS-CA1 resistance for the duration of this 24-week efficacy study.

## Discussion

Long-acting PrEP regimens might overcome some of the current barriers to PrEP implementation, including the need for daily administration and frequent healthcare interaction. The HPTN 083 study recently demonstrated that long-acting ARV monotherapy agents such as CAB-LA can effectively reduce the rate of HIV-1 acquisition^14^. In the present study, we investigated whether a single administration of the capsid inhibitor GS-CA1 could provide long-term protection from repeated SHIV challenges in rhesus macaques. GS-CA1 provided significant protection from rectal infection after 15 repeat challenges, with complete protection achieved when GS-CA1 levels were more than twice the rhesus paEC_95_.

Given the similar structural, mechanistic and long-acting properties of GS-CA1 and LEN, the preclinical GS-CA1 data in this study may inform the clinical development of LEN for PrEP. Our data show protection against rectal SHIV acquisition in macaques at plasma GS-CA1 exposures more than twice the rhesus paEC_95_, which suggests that the current clinical formulation of LEN, conferring a mean exposure of more than six times human paEC_95_ for at least 6 months after a single subcutaneous dose, might be sufficient for PrEP in humans. Nonetheless, the exposure threshold for prophylaxis against HIV acquisition with LEN in humans cannot be directly inferred from this escalating-dose preclinical study and will need to be defined in adequately powered clinical studies. Moreover, animal studies may underestimate exposures required for protection, as evident from the 4 of 12 incident infections among 2,243 analysed participants in the HPTN 083 study that occurred even though the target CAB-LA exposures were predicted preclinically to be efficacious^19,20^. In future work, it will be important to evaluate the efficacy of long-acting capsid inhibitors by other routes of transmission (e.g., vaginal) to establish broader relevance to the populations at risk. Furthermore, a study incorporating mucosal biopsies could establish the pharmacokinetic relationship between plasma and tissue levels of GS-CA1 at the sites of infection.

The emergence of resistance mutations may be an important consideration during the implementation of any single-agent long-acting PrEP strategy. In the phase 3 HPTN 083 trial, 16 HIV seroconversions occurred among participants randomized to CAB-LA, including 4 baseline infections^20^. Genotyping in 14 of these 16 cases revealed integrase mutations among 5 participants, although no resistance was observed among infections presumed to have occurred during the pharmacokinetic tail. Our preclinical study revealed no resistance mutations in the capsid protein among nine rhesus macaques that were treated with GS-CA1 and became infected. However, the low-level resistance mutation Q67H in capsid (conferring a sixfold decrease in susceptibility to LEN^16^) was detected in 2 of 29 participants randomized to long-acting LEN monotherapy in a recent phase 1b study^21^. This mutation was detected 9 days after dose administration in at least one study participant in two of the LEN treatment arms among the five total receiving the lowest doses (20 mg and 50 mg) when the average LEN plasma concentrations measured 0.7 and 1.1 times the human paEC_95_, respectively; both concentrations are considered to be subtherapeutic for HIV-1 treatment. These data suggest that large clinical studies will likely be necessary to fully characterize the potential emergence of resistance mutations among long-acting PrEP recipients.

In summary, our data demonstrate that a single subcutaneous administration of the capsid inhibitor GS-CA1 provides long-term protection against SHIV infection in rhesus macaques. Together with recent studies showing the potency and pharmacokinetics of LEN in people living with HIV, these data suggest that long-acting capsid inhibitors might offer an important and novel strategy for HIV prevention. A phase 3 clinical study to assess the safety and effectiveness of LEN for HIV PrEP has therefore been initiated (NCT04925752).

## Methods

### Drug and formulation

GS-CA1 and a generic internal small-molecule standard used for liquid chromatography coupled with mass spectrometry (LC–MS) experiments were both synthesized at Gilead Sciences and were subjected to standard quality control analysis. For the animal dosing studies, GS-CA1 was dissolved in vehicle (58.03% PEG 300, 27.1% water, 6.78% ethanol, 6.61% poloxamer 188, 1.48% sodium hydroxide) at 300 mg ml^−1^, producing a clear, yellow–orange solution. The solution was stored at ambient temperature protected from light until dosing.

### Metabolic stability of [^3^H]GS-CA1 in primary rhesus hepatocytes

A 500-μl suspension of human hepatocytes (1 × 10^6^ cells per ml) and 0.25 μM [^3^H]GS-CA1 was prepared in Krebs–Henseleit buffer (KHB) medium and was incubated in a humidified incubator at 37 °C with 5% CO_2_ in duplicate wells of a 24-well plate. Propranolol (1 μM final), a compound known to be efficiently metabolized by hepatocytes by oxidation and conjugation, was used as a positive control. A cell-free control was incubated in parallel as a negative control. Aliquots (100 μl) were removed after 0 h, 1 h, 3 h and 6 h and were then mixed with 200 μl quenching solution, placed on a shaker for 10 min and centrifuged at 3,000*g* for 60 min. The supernatant was transferred to a new plate, diluted with 100 μl water and placed on a shaker for 10 min. Quantification of [^3^H]GS-CA1 and its metabolites was performed by radio flow chromatography using a PerkinElmer Radiomatic 625TR flow scintillation analyser with a 500-μl flow cell coupled to a Dionex Ultimate 3000 high-performance liquid chromatography (HPLC) system. PerkinElmer Ultima-Flo was used as the scintillation cocktail, which was mixed with the HPLC effluent at a ratio of 1:1. Sample (100 μl) was injected using a Leap Technologies CTC PAL autosampler. Separation was achieved on a Phenomenex Synergi Fusion-RP 80-Å pore size, 4-μm particle size, 150 × 4.6-mm column maintained at 32 °C. Mobile phase A consisted of 95% water, 5% acetonitrile and 0.1% trifluoroacetic acid (TFA). Mobile phase B consisted of 95% acetonitrile, 5% water and 0.1% TFA. Elution was achieved at a flow rate of 1 ml min^−1^ by linear gradients: the initial condition was 2% B at 0 min, which was increased to 75% B over 45 min, held for 4 min at 75% B and then returned to the initial conditions. The column was allowed to re-equilibrate for 12 min between injections. Quantification was based on the radiochromatographic peak area using Dionex (Thermo Scientific) Chromeleon 6.8 software.

### Cell culture

Freshly isolated PBMCs from three male rhesus macaque (*M. mulatta*) donors of Indian origin (HumanCells Biosciences) were cultured in RPMI-1640 cell culture medium (Life Technologies) supplemented with 10% heat-inactivated FBS (Hyclone), 2 mM glutamine and 100 U ml^−1^ penicillin plus 100 μg ml^−1^ streptomycin (complete RPMI). Before their use in the antiviral assays, rhesus PBMCs from three independent donors were pooled and activated at a density of 3 × 10^6^ cells per ml for 72 h at 37 °C by the addition of 1 μg ml^–1^ phytohemagglutinin (PHA, Sigma-Aldrich) and 50 U ml^−1^ recombinant human interleukin-2 (IL-2) (Roche Diagnostics).

### Anti-SHIV antiviral assay in rhesus PBMCs

PHA/IL-2-stimulated rhesus PBMCs were infected in bulk culture with SHIV-SF162P3 at a concentration of 130 pg of p27 equivalent per million PBMCs. The cells were maintained in suspension by gently rocking the cultures mixed with virus inoculum for 3 h at 37 °C. The cells were then pelleted by centrifugation at 500*g* for 5 min, washed twice with complete RPMI to remove any unadsorbed virus and seeded into 96-well plates at a cell density of 2 × 10^5^ cells per well in 100 μl. Eight-point threefold serial dilutions of GS-CA1 were made in complete RPMI containing 50 U ml^−1^ IL-2 and were added in triplicate to wells containing cells (100 μl per well). The cultures were incubated in a 5% CO_2_ incubator at 37 °C for 7 days. Cell-free supernatants derived from the PBMC cultures were harvested 7 days after infection, the amount of SHIV present was quantified using an SIV p27 antigen-capture ELISA assay (5436, Advanced Bioscience Laboratories) performed according to the manufacturer’s protocol, and the data were acquired using SoftMax Pro 6.3.1 software (Molecular Devices). The mean EC_50_ value for GS-CA1, which was determined from a total of seven assays performed in triplicate, was calculated from the dose–response curves using XLfit 5.5.0.5 software (IDBS) and was expressed graphically using GraphPad Prism 8.1.2 software. The Hill coefficient (*n*) for GS-CA1 was measured from the slope of the dose–response curves (*n* = 3.03 ± 0.69) and was used to derive the EC_95_ value using the following equation: EC_95_ = EC_50_ × (95/5)^1/*n*^.

### Equilibrium dialysis shift assay

Rhesus plasma protein binding to GS-CA1 was determined by competitive equilibrium dialysis. Rhesus plasma (10%) was spiked with GS-CA1 (2 μM), and this mixture and blank RPMI cell culture medium containing 10% FBS (CCM) were placed into opposite sides of assembled dialysis cells; afterwards, incubations were performed in triplicate. After a 24-h equilibration period at 37 °C, samples were quenched with 4 volumes of 90% (vol/vol) acetonitrile and 10% (vol/vol) methanol containing internal standard. Then, they were quantified using the AB Sciex API 4000 LC–MS/MS system (GenTech Scientific) with electrospray ionization in positive mode and multiple-reaction monitoring and were analysed using Analyst 1.6.1 software. The fold change value in 100% rhesus plasma was then calculated using the plasma/CCM ratio after correcting for the sample dilution factor and the percentage of free fraction in the matrix. This shift value was multiplied by the calculated anti-SHIV EC_95_ value for GS-CA1 to derive the corresponding rhesus plasma paEC_95_.

### Animals, drug administration and viral stocks

Studies involving the evaluation of GS-CA1 pharmacokinetics in naive male rhesus macaques of Indian origin were conducted at Covance Laboratories in a Laboratory Animal Care-accredited facility and were performed in strict compliance with all relevant ethical regulations. All study protocols were reviewed and approved by the Covance Laboratories Institutional Animal Care and Use Committee (IACUC). On day 0 of the study, rhesus monkeys were administered GS-CA1 dosed at 100 mg kg^−1^ (*n* = 2) or 300 mg kg^−1^ (*n* = 3) in the scapular region by subcutaneous injection using a syringe equipped with a 22-gauge needle. The GS-CA1 was prepared in a stock solution of 300 mg ml^−1^; a maximum of 2 ml of the solution was injected into a single subcutaneous site. Whole blood was collected from each animal at designated time points, processed into plasma and then stored frozen at −80 °C for bioanalysis of GS-CA1 levels.

For the challenge study, 24 outbred rhesus macaques of Indian origin were assigned to the three study groups with even sex and weight distributions. All animals were housed at Alpha Genesis (Yemassee, SC), and all procedures were conducted in compliance with all relevant local, state and federal regulations and were approved by the Alpha Genesis IACUC. On week 0, the three groups were administered vehicle control, GS-CA1 dosed at 300 mg kg^−^^1^ or GS-CA1 dosed at 150 mg kg^−1^ in the scapular region by subcutaneous injection using a syringe equipped with a 22-gauge needle. The GS-CA1 was prepared in a stock solution of 300 mg ml^−1^; a maximum of 2 ml of solution was injected into a single subcutaneous site. The injection sites were monitored daily by veterinary staff for 3 days. Beginning in week 1, animals in all three groups were challenged by the intrarectal route with 1 ml of RPMI containing the specified dilution of a 3.62 × 10^3^ TCID_50_ SHIV-SF162P3 stock. At each respective time point, the challenges were performed on the same day, using the same virus stock and inoculation method across the three groups. Whole blood was collected and processed into plasma and PBMCs as necessary to assess routine haematology, clinical chemistry, viral load and serology as well as for bioanalysis of drug levels. The animals were considered to be protected if they remained SHIV negative by plasma PCR assay and seronegative by enzyme immunoassay throughout the 15-week challenge phase and the 9-week follow-up.

### Bioanalysis of GS-CA1 in macaque plasma

Rhesus plasma samples were stored frozen at −80 °C. For analysis, the samples were thawed, and a 50-µl aliquot of each sample was treated with 200 µl acetonitrile containing a generic internal small-molecule standard. After precipitation of the protein component, a 100-µl aliquot of the supernatant was transferred to a clean 96-well plate and was mixed with 200 µl water. A 20-μl aliquot of the above solution was then injected into a Q-Exactive high-resolution mass spectrometer (Thermo Scientific) with electrospray ionization in positive mode. Quantification was performed with a Thermo Scientific Xcalibur 4.0.27.19 using accurate masses (<5 parts per million mass error; [M + H]^+^ of 958.1853 for GC-CA1 and 758.3270 for the internal standard, respectively)^22,23^. The lower and upper limits of quantification for GS-CA1 were 1 nM and 10,000 nM, respectively. Pharmacokinetic parameters, including the area under the plasma concentration–time curve from time 0 to the last quantifiable time point (AUC_last_), the area under the plasma concentration–time curve from time 0 to infinity (AUC_inf_), the maximum concentration (*C*_max_), the time to reach the observed peak plasma concentration (*t*_max_) and the terminal half-life (*t*_1/2_), were determined by non-compartmental analysis using Phoenix WinNonlin 6.4 build 8.1.0.3530 software (Pharsight).

### Plasma viral load assay

RNA was extracted from rhesus macaque plasma using a QIAcube high-throughput instrument and an IndiSpin QIAcube HT Pathogen kit (Qiagen). RNA standards using the SIV *gag* sequence were generated by AmpliCap-Max T7 High-Yield Message Maker kit (Cell Script) and were purified using an RNA Clean and Concentrator kit (Zymo Research). Log dilutions of the RNA were included with each RT–qPCR assay. Reverse transcription of standards and samples was performed using a Superscript III VILO kit (Invitrogen). qPCR was performed using forward primer 5′-GTCTGCGTCATCTGGTGCATTC-3′, reverse primer 5′-CACTAGGTGTCTCTGCACTATCTGTTTTG-3′ and fluorescently labelled primer 5′-CTTCCTCAGTGTGTTTCACTTTCTCTTCTGCG-3′ on a Quantstudio 6 Flex system (Applied Biosystems). The assay LOD was 200 copies *gag* per ml.

### ELISA

Rhesus serum binding antibody titres against gp140 trimers were determined by ELISA. Ninety-six-well Maxisorp ELISA plates (Thermo Fisher Scientific) were coated overnight with 100 μl per well of 1 μg ml^−1^ mosaic M gp140 protein in PBS and were then blocked for 2 h with blocker casein in PBS (Thermo Scientific). Macaque sera were added in threefold serial dilutions and were incubated for 1 h at room temperature. The plates were washed three times with PBS containing 0.05% Tween-20 and were then incubated for 1 h with a 1:1,000 dilution of a horseradish peroxidase (HRP)-conjugated goat anti-human secondary antibody (Jackson ImmunoResearch Laboratories). The plates were then washed three times with the above wash buffer and were developed using SureBlue tetramethylbenzidine (TMB) microwell peroxidase (KPL). Development was stopped by the addition of a stop solution (KPL), and the plates were then analysed at 450 nm with a Versamax ELISA microplate reader (Molecular Devices) using Softmax Pro 6.5.1 software. The ELISA endpoint titres were defined as the highest reciprocal serum dilution that yielded an OD_450_ _nm_ absorbance of >0.2. Seroconversion occurred when the OD_450_ _nm_ absorbance was greater than or equal to three times the value obtained at week 0 and the endpoint titre was greater than the assay LOD, which was 1:25.

### IFNγ ELISPOT assay

T cell responses against the Gag polyprotein were determined in rhesus macaque PBMCs by ELISPOT assay. Ninety-six-well Multiscreen Immobilon-P plates (Millipore) were coated overnight with mouse anti-human interferon γ (IFNγ) antibody (BD Pharmingen) at 5 μg ml^−1^. The plates were then washed three times with DPBS containing 0.25% Tween-20 and blocked with R10 (RPMI supplemented with 10% FBS and 1% penicillin–streptomycin) at 37 °C for 1 h. SIVmac239-derived Gag peptides (JPT) were plated at a concentration of 1 µg per well along with 200,000 rhesus macaque cells per well, and the cells and peptides were then incubated for 18–24 h at 37 °C. All subsequent steps were performed at room temperature. The wells were washed nine times with the above wash buffer, cells were lysed with deionized water for 3 min and plates were then incubated with biotinylated rabbit anti-human IFNγ antibody (U-Cytech) at a final concentration of 1 μg ml^−1^ for 2 h. The wells were then incubated with streptavidin–alkaline phosphatase (Southern Biotech) at a final concentration of 2 μg ml^−1^ for 2 h, washed five times with the above wash buffer and incubated with 5-bromo-4-chloro-3′-indolyphosphate *p*-toluidine salt (BCIP)/nitro-blue tetrazolium chloride (NBT) substrate solution (Thermo Scientific Pierce) for 7 min. The spot-forming cell (SFC) units were sent to ZellNet Consulting for quantification. The mock-stimulated background-subtracted median values were reported, and the assay LOD was 5 SFCs per 10^6^ PBMCs.

### Intact proviral DNA assay

An SHIV-adapted version of IPDA (SHIV-IPDA) was used to determine the number of intact SHIV proviruses. Total genomic DNA was extracted from unfractionated PBMCs using a QIAamp DNA Mini kit (Qiagen). DNA quality and quantity were evaluated by spectrophotometry and fluorometry, respectively, and SHIV-IPDA was then performed on the isolated DNA. An in-depth description of SHIV-IPDA will be included in an upcoming manuscript by E. Fray et al. In brief, SHIV-IPDA consists of a three-component multiplex droplet digital PCR (ddPCR) reaction. The first is a SHIV proviral discrimination reaction targeting two conserved, frequently deleted regions of the SHIV genome to determine the intact provirus count; the second is a two-long terminal repeat (2-LTR) DNA circle reaction to determine 2-LTR circle counts; and the third is a copy reference/DNA shearing reaction targeting ribonuclease P/MRP subunit P30 (RPP30) to determine assay input cell equivalents and the DNA shearing index (DSI). All ddPCR reactions were performed using a Bio-Rad QX200 AutoDG ddPCR system with Bio-Rad ddPCR supermix for probes with no dUTP. After DSI correction and subtraction of intact 2-LTR circles, the intact proviral frequencies were reported per million input cells. The endpoint ddPCR data were collected using Bio-Rad QuantaSoft version 1.7.4.0917.

### Plasma virus genotypic analysis

Total RNA was extracted from 50-µl plasma aliquots obtained from each viraemic monkey using the MagMAX-96 Viral RNA Isolation kit (Life Technologies) in conjunction with the Thermo Scientific KingFisher Flex automated extraction platform and were eluted in 60 μl AVE buffer. The portion of *gag* encoding capsid in each sample was then individually amplified by RT–PCR using a SuperScript IV One-Step RT–PCR System (Life Technologies) according to the manufacturer’s recommended protocol. Amplification of the SHIV capsid-encoding region in each sample was performed using primers SIV-CA-F (5′-CCAAAAACAAGTAGACCAACAG-3′) and SIV-CA-R (5′-TGCAAAAGGGATTGGCAC-3′), and the products were subjected to population-level bulk sequencing at Elim Biopharmaceuticals using the same primer set. To identify codon changes, Sequencher version 4.9 (Gene Codes) was used to align DNA encoding sequences of the SHIV capsid for each sample with that of the parent virus obtained from each infected placebo-treated control animal. This provided a control for potential genetic drift that might have occurred during the 24-week efficacy study.

### Statistical methods

Protection against acquired infection was analysed using Cox proportional hazard regression models based on the exact partial likelihood for discrete time expressed in weeks, which explicitly accounts for the time-varying viral challenge dose in the model. The hazard ratios with 95% confidence intervals for per-exposure relative reductions in acquisition risk were calculated for the GS-CA1-treated groups, and the results were compared with those for the vehicle-treated control group. Comparisons were considered statistically significant at a two-sided alpha level of 0.05 (*P* < 0.05). Statistical analyses were performed using GraphPad Prism version 8.1.2, SAS version 9.4 and R Studio software version 4.0.5.

### Reporting summary

Further information on research design is available in the Nature Research Reporting Summary linked to this paper.

## Online content

Any methods, additional references, Nature Research reporting summaries, source data, extended data, supplementary information, acknowledgements, peer review information; details of author contributions and competing interests; and statements of data and code availability are available at 10.1038/s41586-021-04279-4.

### Supplementary information

Reporting Summary

### Source data

Source Data Fig. 1

Source Data Fig. 2

Source Data Fig. 3

## Figures and Tables

**Extended Data Table 1:**
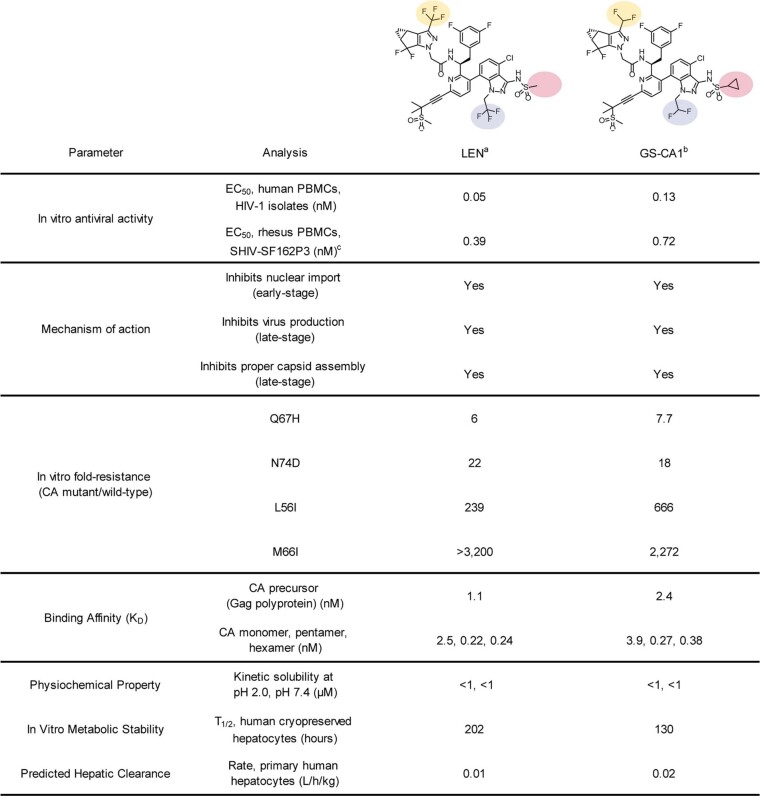
Comparison of the major structural and functional attributes for small molecule capsid inhibitors LEN and GS-CA1

a, Link et al, Nature, 2020;^16^ b, Yant et al, Nat.Med, 2019;^4^ c, this report. Colored regions highlight structural distinctions between these long-acting capsid inhibitors.

**Extended Data Table 2:**
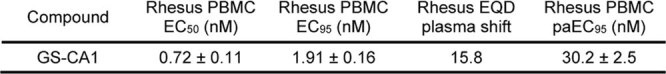
Determination of the GS-CA1 rhesus paEC_95_ in rhesus PMBCs

The GS-CA1 EC_50_ against SHIV-SF162P3 in rhesus PMBCs calculated from dose-response curves, the estimated EC_95_ based on the Hill slope value against the HIV-1 IIIb strain in MT-4 cells, and the rhesus protein adjusted EC_95_ (paEC_95_) based on competitive equilibrium dialysis (EQD) plasma shift assays. EC_50_ data (mean ± s.d.) are from 7 independent assays (n = 3 biological replicates each). EQD data are mean values from three independent experiments performed in singlet.

**Extended Data Table 3:**
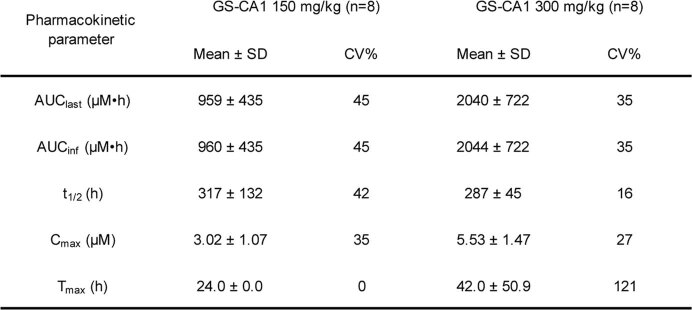
Pharmacokinetic parameters following a single subcutaneous administration of GS-CA1 in rhesus macaques

Pharmacokinetic parameters, including area under the plasma concentration–time curve from time 0 to the last quantifiable time point (AUC_last_), area under the plasma concentration–time curve from time 0 to infinity (AUC_inf_), maximal concentration (C_max_), time to reach observed peak plasma concentration (T_max_), and terminal half-life (t_1/2_) determined by non-compartmental analysis using Phoenix WinNonlin 6.4 build 8.1.0.3530. CV, coefficient of variation (100 X standard deviation / mean).

**Extended Data Table 4:**
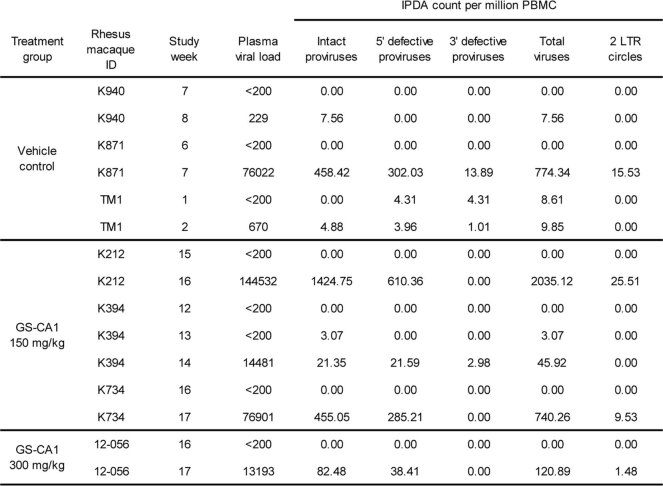
Detection of intact proviral SHIV DNA relative to viremia in the challenge study

SHIV proviral DNA counts among peripheral blood mononuclear cells (PBMCs) determined by intact proviral DNA assay (IPDA) in infected rhesus macaques treated with vehicle control (n = 3), GS-CA1 dosed at 150 mg/kg (n = 3), or GS-CA1 dosed at 300 mg/kg (n = 1) at the indicated timepoints. SHIV viremia measured by *gag* RT-qPCR in the same animals and timepoints and reported as SHIV RNA copies per ml.

**Extended Data Table 5:**
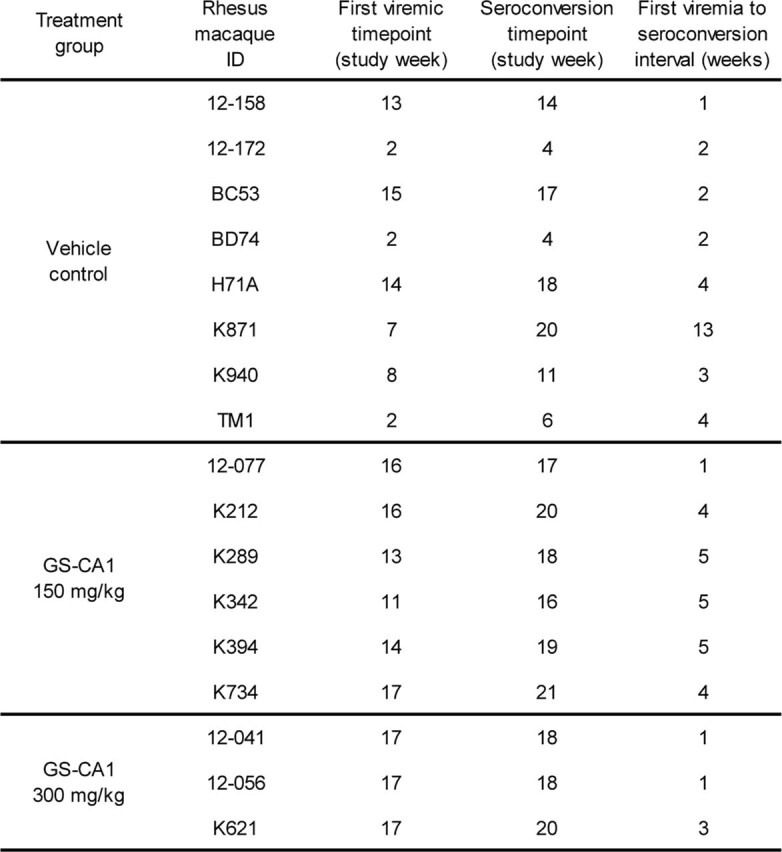
Time from onset of viremia to seroconversion among infected rhesus macaques treated with vehicle control or GS-CA1

Onset of plasma SHIV viremia measured by *gag* RT-qPCR in the three study groups. Seroconversion timepoints determined by serum Env ELISA among infected animals in the three groups.

## Data Availability

All relevant data are available in the Article. Any additional data are available from the corresponding authors upon reasonable request. Source data are provided with this paper.
