# Supplementary material for: Long-acting capsid inhibitor protects macaques from repeat SHIV challenges
Source: Nature. 2021 Dec 7;601(7894):612–6. doi: 10.1038/s41586-021-04279-4 (PMC8753592; doi:10.1038/s41586-021-04279-4)
Supplement: Supplementary file 1 — Reporting Summary [file 41586_2021_4279_MOESM1_ESM.pdf]

nature portfolio

Corresponding author(s): Dan Barouch

Last updated by author(s): Nov 18, 2021

# Reporting Summary

Nature Portfolio wishes to improve the reproducibility of the work that we publish. This form provides structure for consistency and transparency in reporting. For further information on Nature Portfolio policies, see our Editorial Policies and the Editorial Policy Checklist.

## Statistics

For all statistical analyses, confirm that the following items are present in the figure legend, table legend, main text, or Methods section.

| n/a | Confirmed | |
|---|---|---|
| ☐ | ☒ | The exact sample size (*n*) for each experimental group/condition, given as a discrete number and unit of measurement |
| ☐ | ☒ | A statement on whether measurements were taken from distinct samples or whether the same sample was measured repeatedly |
| ☐ | ☒ | The statistical test(s) used AND whether they are one- or two-sided<br>*Only common tests should be described solely by name; describe more complex techniques in the Methods section.* |
| ☒ | ☐ | A description of all covariates tested |
| ☒ | ☐ | A description of any assumptions or corrections, such as tests of normality and adjustment for multiple comparisons |
| ☐ | ☒ | A full description of the statistical parameters including central tendency (e.g. means) or other basic estimates (e.g. regression coefficient) AND variation (e.g. standard deviation) or associated estimates of uncertainty (e.g. confidence intervals) |
| ☐ | ☒ | For null hypothesis testing, the test statistic (e.g. *F*, *t*, *r*) with confidence intervals, effect sizes, degrees of freedom and *P* value noted<br>*Give P values as exact values whenever suitable.* |
| ☒ | ☐ | For Bayesian analysis, information on the choice of priors and Markov chain Monte Carlo settings |
| ☒ | ☐ | For hierarchical and complex designs, identification of the appropriate level for tests and full reporting of outcomes |
| ☒ | ☐ | Estimates of effect sizes (e.g. Cohen's *d*, Pearson's *r*), indicating how they were calculated |

*Our web collection on statistics for biologists contains articles on many of the points above.*

## Software and code

Policy information about availability of computer code

| | |
|---|---|
| Data collection | SoftMax Pro 6.3.1 was used to acquire SIV p27 ELISA data.<br>Thermo Scientific Xcalibur 4.0.27.19 was used to collect LC/MS data.<br>Viral load data were collected using the Quantstudio version 6 Flex Real-Time PCR System (Applied Biosystems).<br>Endpoint droplet digital PCR data were collected using Bio-Rad QuantaSoft version 1.7.4.0917. |
| Data analysis | XLfit 5.5.0.5 software (IDBS) was used to analyze SIV p27 ELISA data and plot data for antiviral dose response curves.<br>Analyst 1.6.1 software was used to analyze LC/MS-MS equilibrium dialysis shift data.<br>Dionex (Thermo Scientific) Chromeleon 6.8 software was used to analyze radiochromatographic peak area data for [3H]GS-CA1 and its metabolites.<br>GraphPad Prism version 8.1.2 was used to prepare Kaplan-Meier animal infection rate plots, with Cox regression analysis performed using SAS version 9.4 and R Studio version 4.0 softwares.<br>Thermo Scientific Xcalibur 4.0.27.19 was used to analyze LC/MS and high-resolution mass spectrometry data for plasma GS-CA1 levels.<br>Phoenix WinNonlin 6.4 build 8.1.0.3530 was used for noncompartmental analyses of pharmacokinetic (PK) parameters.<br>SHIV capsid coding region sequencing analysis used Sequencher version 4.9. |

For manuscripts utilizing custom algorithms or software that are central to the research but not yet described in published literature, software must be made available to editors and reviewers. We strongly encourage code deposition in a community repository (e.g. GitHub). See the Nature Portfolio guidelines for submitting code & software for further information.

## Data

Policy information about availability of data

All manuscripts must include a data availability statement. This statement should provide the following information, where applicable:

- Accession codes, unique identifiers, or web links for publicly available datasets
- A description of any restrictions on data availability
- For clinical datasets or third party data, please ensure that the statement adheres to our policy

All relevant data are available in the article. Any additional data are available from the corresponding authors upon reasonable request. Source data are provided with this paper.

# Field-specific reporting

Please select the one below that is the best fit for your research. If you are not sure, read the appropriate sections before making your selection.

☒ Life sciences ☐ Behavioural & social sciences ☐ Ecological, evolutionary & environmental sciences

For a reference copy of the document with all sections, see nature.com/documents/nr-reporting-summary-flat.pdf

# Life sciences study design

All studies must disclose on these points even when the disclosure is negative.

| | |
|---|---|
| Sample size | We did not rely on statistical analyses to determine sample size since we could not estimate a priori the level of protection which may be afforded by a long acting capsid inhibitor, a new class of HIV antiviral. However, we modeled our design after repeat challenge non-human primate studies previously conducted by us and other groups evaluating other classes of HIV antivirals for pre-exposure and post-exposure prophylaxis. |
| Data exclusions | No data were excluded. |
| Replication | Virologic and immunologic measures were performed in duplicate. Technical replicates were minimally different. All attempts at replication were successful. |
| Randomization | Animals were balanced for age and gender and otherwise randomly allocated to groups. |
| Blinding | All immunologic and virologic assays were performed blinded. |

# Reporting for specific materials, systems and methods

We require information from authors about some types of materials, experimental systems and methods used in many studies. Here, indicate whether each material, system or method listed is relevant to your study. If you are not sure if a list item applies to your research, read the appropriate section before selecting a response.

## Materials & experimental systems

| n/a | Involved in the study | |
|---|---|---|
| ☐ | ☒ | Antibodies |
| ☒ | ☐ | Eukaryotic cell lines |
| ☒ | ☐ | Palaeontology and archaeology |
| ☐ | ☒ | Animals and other organisms |
| ☒ | ☐ | Human research participants |
| ☒ | ☐ | Clinical data |
| ☒ | ☐ | Dual use research of concern |

## Methods

| n/a | Involved in the study | |
|---|---|---|
| ☒ | ☐ | ChIP-seq |
| ☒ | ☐ | Flow cytometry |
| ☒ | ☐ | MRI-based neuroimaging |

## Antibodies

| | |
|---|---|
| Antibodies used | For evaluation of the GS-CAI EC50 against SHIV-SF162P3 in rhesus macaque PBMCs, SIV p27 antigen capture ELISA assay (Advanced Bioscience Laboratories, catalog number 5436) was used. For detection of rhesus macaque humoral responses to SHIV-SF162P3 Env, ELISA was used with horseradish peroxidase (HRP)-conjugated goat anti-human secondary antibody (Jackson ImmunoResearch Laboratories, catalog number 109-035-003). For detection of rhesus macaque cellular responses to SHIV-SF162P3 Gag, ELISPOT was used with mouse anti-human IFN-γ antibody (BD Pharmingen, catalog number 554699) and biotinylated rabbit anti-human IFN-γ (U-CyTech biosciences, catalog number CT243). |

| | |
|---|---|
| Validation | Antibodies were used according to manufacturer's instructions and websites as well as previously published methods for all assays. Antibodies were validated and titrated for specificity prior to use for all assays. |

## Animals and other organisms

Policy information about studies involving animals; ARRIVE guidelines recommended for reporting animal research

| | |
|---|---|
| Laboratory animals | 24 outbred Indian-origin male and female rhesus macaques (Macaca mulatta), 3-6 years old |
| Wild animals | None |
| Field-collected samples | None |
| Ethics oversight | All studies were performed in accordance with the Covance and Alpha Genesis Inc. Institutional Animal Care and Use Committees |

Note that full information on the approval of the study protocol must also be provided in the manuscript.
